# Influence of *SERPINA1* Gene Polymorphisms on Anemia and Chronic Obstructive Pulmonary Disease

**DOI:** 10.1155/2022/2238320

**Published:** 2022-10-17

**Authors:** Thangavelu Sangeetha, Tajuddin Nargis Begum, Balasubramanian Balamuralikrishnan, Meyyazhagan Arun, Kannan R. R. Rengasamy, Natchiappan Senthilkumar, Shanmugam Velayuthaprabhu, Muthukrishnan Saradhadevi, Palanisamy Sampathkumar, Arumugam Vijaya Anand

**Affiliations:** ^1^Department of Human Genetics and Molecular Biology, Bharathiar University, Coimbatore, Tamil Nadu, India; ^2^Department of Biotechnology, Jamal Mohammed College, Tiruchirapalli, Tamil Nadu, India; ^3^Department of Food Science and Biotechnology, Sejong University, Seoul, Republic of Korea; ^4^Department of Life Sciences, Christ Deemed to be University, Bengaluru, India; ^5^Laboratory of Natural Products and Medicinal Chemistry (LNPMC), Department of Pharmacology, Saveetha Dental College, Saveetha Institute of Medical and Technical Sciences (SIMATS), Chennai, Tamil Nadu 600077, India; ^6^Centre of Excellence for Pharmaceutical Sciences, North-West University, Potchefstroom 2520, South Africa; ^7^Chemistry and Bioprospecting Division, Institute of Forest Genetics and Tree Breeding (IFGTB-ICFRE), Coimbatore, Tamil Nadu, India; ^8^Department of Biotechnology, Bharathiar University, Tamil Nadu, India; ^9^Department of Biochemistry, Bharathiar University, Tamil Nadu, India; ^10^Department of Chemistry and Biosciences, SASTRA Deemed to be University, Kumbakonam, Tamil Nadu, India

## Abstract

**Background:**

Anemia is one of the predominant hematological conditions, whereas chronic obstructive pulmonary disease (COPD) is a predominant respiratory disease. These two diseases were found to be interlinked, but the physiological pathways are still unclear.

**Aim:**

The current study has been aimed at analysing the genetic interrelationship between anemia and COPD in accordance with different altitudes. *Methodology*. The genetic analysis was performed in the *SERPINA1* gene of anemia, COPD, and healthy individuals for the analysis of single nucleotide polymorphism at rs28949274 and rs17580 locations. *Result and Discussion*. The single nucleotide polymorphism at the locations rs28949274 and rs17580 was present in both anemic and COPD patients. The COPD patients were more prone to mutations (63% had rs28949274, and 11% had rs17580 polymorphisms) than the anemic patients (40% had rs28949274, and 1% had rs17580 polymorphisms). On the basis of altitude, high-altitude individuals were found to be more susceptible to both the polymorphisms.

**Conclusion:**

Based on the current findings, we suggest that the *SERPINA1* gene has a positive correlation with anemia as well as COPD, and the increase in altitude also influences the diseased conditions in a positive manner.

## 1. Introduction

Anemia is a predominant clinical condition in which the concentration of hemoglobin or the erythrocyte count is lower than normal [1, 2], which affects more than 33.3% of the global population [3–5]. The etiology of anemia varies from diet, like insufficient or lack of intake of iron-containing foods, to environmental factors like altitude, including the genetic makeup of the individual [6]. A respiratory condition, which is characterised by airflow obstructions, has been termed chronic obstructive pulmonary disease (COPD), which accounts for about 1% of the global population irrespective of age and rises above 10% in the population over 40 years of age [7]. The causative agents of COPD include major lifestyle habits like smoking and exposure to toxic gases and other factors like genetic polymorphisms [8].

**Figure 1:**
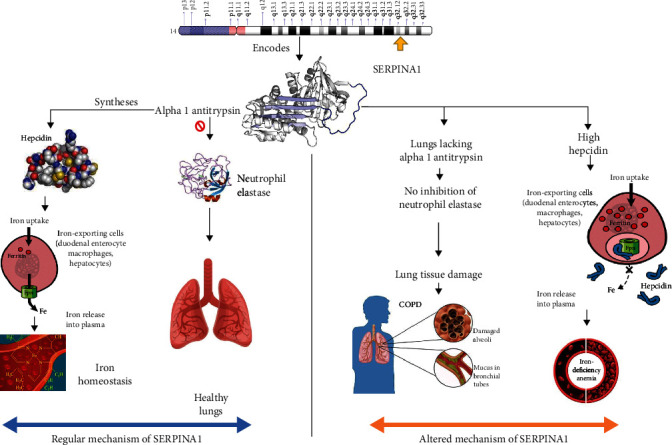
Pathophysiology of *SERPINA1* gene in anemia and COPD.

The genetic basis of anemia and COPD is being investigated for their interrelationship on the genetic basis. *SERPINA1* encodes a protein called alpha 1 antitrypsin, which is involved in the management of neutrophil elastase enzyme released at the site of inflammation as well as hepcidin hormone, which is involved in iron homeostasis [9, 10]. The pathophysiology of the *SERPINA1* gene in anemia and COPD has been given in Figure 1. The current study has been aimed at analysing the genetic interrelationship of the *SERPINA1* gene with anemia and COPD in comparison with the control samples by analysing the whole sequence of the gene and at the locations rs28929474 and rs17580 for the presence or absence of single nucleotide polymorphism.

## 2. Materials and Methodology

### 2.1. Sample Collection

**Figure 2:**
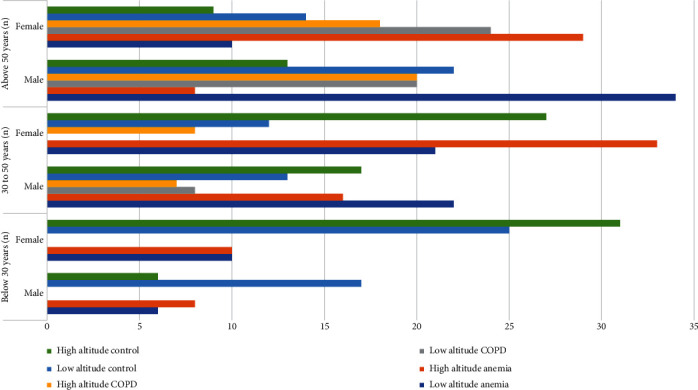
Sample categorisation based on age factor.

The sample collection (*N* = 518) and categorisation have been given in Table 1 and Figure 2. After obtaining the proper consent form, 2 mL of human blood has been collected from anemic, COPD patients, and healthy individuals after obtaining the proper consent form by using the venipuncture method. A human ethical clearance certificate has been obtained from the Avinashilingam Deemed University for Women, Coimbatore, Tamil Nadu, India (approval no.: AUW/IHEC–18–19/HGMB/FHP–21). An informed consent form has been obtained from all the subjects before collecting the blood sample.

The current research work involves one-time sample collection, and no follow-up study has been performed. A total of 877 questionnaires (*N* = 877) have been collected from random individuals in low and high-altitude regions and, based on the inclusion and exclusion criteria, the sample collection was performed. The inclusion criteria for anemia samples are hemoglobin range lower than 12.0 g/dL; individuals residing in the areas in and around Coimbatore and The Nilgiris (Districts of Tamil Nadu, South India); age factors between 15 and 65 years; and for COPD samples, individuals who are diagnosed with this respective clinical condition from the areas in and around Coimbatore and The Nilgiris (Southern Districts of Tamil Nadu, South India) have been considered. Pregnant women and children less than 15 years of age are excluded from the sample collection. Based on the questionnaire analysis, the individuals were classified into anemic (based on hemoglobin count), COPD (based on lung function test score), and control (based on absence of any clinical disease or condition).

### 2.2. DNA Isolation and Quantification

The genomic DNA has been isolated from the collected samples (*N* = 518) by using the modified Miller et al. [11] method. The isolation involved the removal of red blood cell debris by using saline and hypotonic solutions containing sucrose, magnesium chloride, and triton X, followed by the removal of protein complexes by sodium lauryl sulphate, sodium perchlorate, and chilled chloroform. The isolated DNA has been stored in 1X TE (tris EDTA) buffer at 4°C until further use. All the chemicals used for the DNA isolation have been obtained from Himedia Laboratories Pvt. Ltd., Mumbai, India. To ensure the purity of the double-stranded DNA, DNA was quantified in Biodrop 2000 at 260/280 nm.

The presence of isolated DNA was confirmed by agarose gel electrophoresis in 1% agarose gel. The absorbance values of samples measured at 260/280 nm ranged from 1.6 to 1.9, showing that the product was pure and perfect for a polymerase chain reaction.

### 2.3. Mutational Analysis

The primers for the whole sequence of the *SERPINA1* gene and for the analysis of single nucleotide polymorphism at the locations rs28929474 and rs17580 have been designed by using the bioinformatics tool and will be used for the mutational analysis. The primer details and the annealing conditions have been given in Table 2. The pure DNA obtained is subjected to polymerase chain reaction in the thermocycler at the specified conditions, and the amplified products are confirmed by performing agarose gel electrophoresis in a 1.5% agarose gel. Single pass sequencing was performed on each template using the below 16 s rRNA universal primers. The fluorescent-labeled fragments were purified from the unincorporated terminators with an ethanol precipitation protocol. The samples were resuspended in distilled water and subjected to electrophoresis on an ABI 3730xl sequencer (Applied Biosystems).

## 3. Results

### 3.1. Mutational Analysis

**Figure 3:**
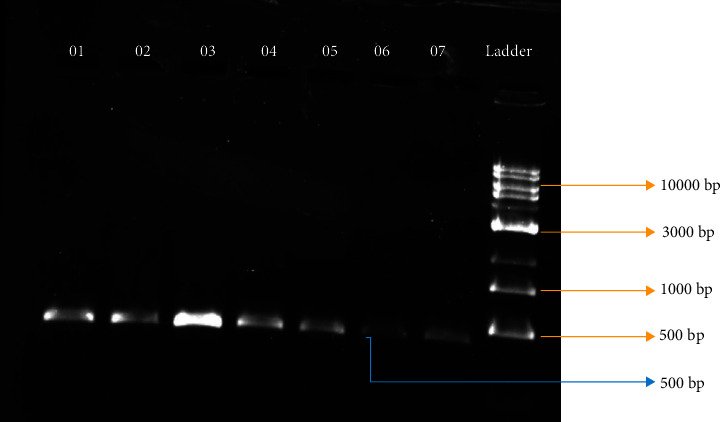
Amplicons obtained using nonmutation specific primer.

**Figure 4:**
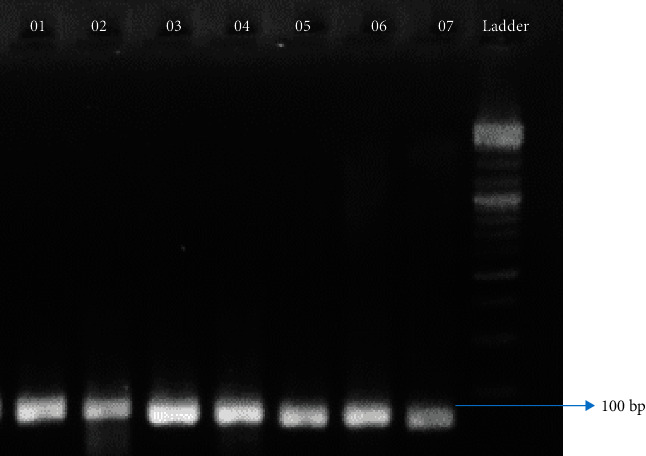
Amplicons obtained during amplification at the location rs28929474.

**Figure 5:**
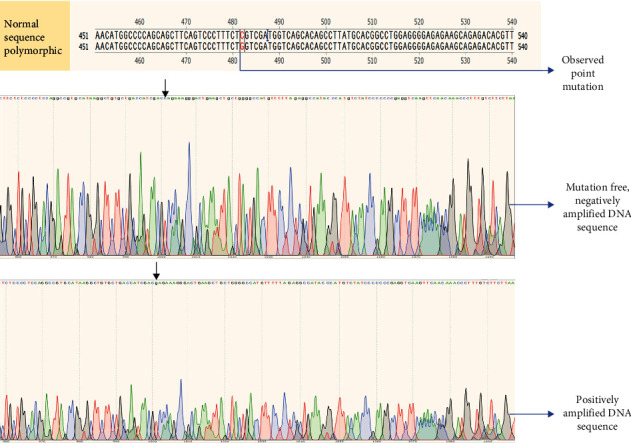
Gene sequencing of the obtained amplicons (location—rs28929474).

Among all the collected samples (*N* = 518), around 55% (*N* = 288) showed positive amplification (Figure 3), with an amplicon size of around 500 bp, which corresponds to 35% anemic (*N* = 73), 11% COPD (*N* = 12), and 98% control (*N* = 203) samples. The negatively amplified samples (*N* = 230) have been then subjected to polymorphic analysis using the mutation-specific primer designed for the location rs28929474 at the specific polymerase chain reaction conditions. The amplicon size was approximately 100 bp, and the presence of products has been confirmed by agarose gel electrophoresis in 1.5% gel (Figure 4). Positive amplification, indicating the presence of single nucleotide polymorphism, was found in 65% (*N* = 150) of the samples tested, including 40% anemic (*n* = 83) and 63% COPD (*n* = 67) samples from both altitudes. Ser482try polymorphism that alters the serine to tryptophan (S→W) amino acid has been observed in the positively amplified samples when the samples are sequenced (Figure 5).

**Figure 6:**
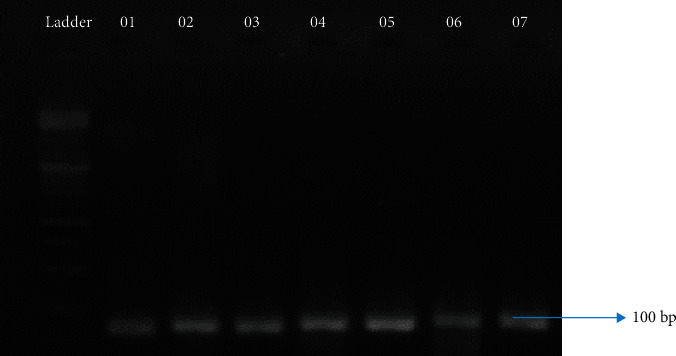
Amplicons obtained during amplification at the location rs17580.

**Figure 7:**
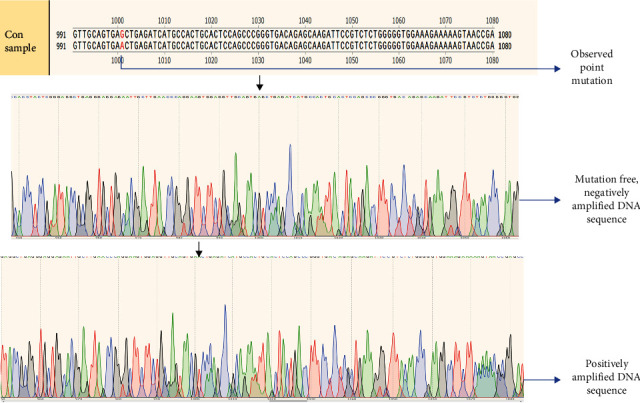
Gene sequencing of the obtained amplicons (location—rs17580).

Followed by rs28929474, the other location rs17580 has been analysed with the remaining samples (*N* = 80), which did not show positive amplification with primers for rs28929474. The amplified products, on examination in 1.5% gel, showed up at around 100 bp in size (Figure 6). Only 2.7% of the total collected samples, i.e., *N* = 14, which accounts for approximately 17% of the analysed samples, showed positive amplification, which comprises approximately 1% anemic (*N* = 2) and 11% COPD (*N* = 12) samples from both altitudes. A nucleotide variation of ser1001asn has been observed that changes the amino acid synthesis from serine to asparagine (S→N) on gene sequencing (Figure 7).

Table 3 shows the altitudinal comparison of anemic, COPD, and control individuals on the basis of positive and negative amplification. The presence of polymorphism has been observed to be higher in high altitude patients of both the diseases when compared to the low altitude patients of the respective clinical conditions.

## 4. Discussion

The present study has correlated anemic conditions with COPD on a genetic basis in a positive manner. Anemia may be caused due to a lack of nutritional intake such as iron, vitamin A, B_6_, B_12_, folate, copper, and zinc. Nutritional deficiency anemia is the most common type of anemia [6, 12]. Aside from dietary factors, anemia can be caused by genetic and epigenetic factors, and it has been classified into a few common types, which include sickle cell anemia and beta-thalassemia, which are caused by alterations in the *HBB* gene; and glucose-6-phosphate dehydrogenase deficiency (G6PD), which is caused by a polymorphic X chromosome [13–15]. The sickle cell anemic patients analysed for the genetic alterations in the *SERPINA1* gene showed a negative association [16].

Anemia in COPD has been found to be more common and, comparatively, the etiology has been found to be iron deficiency [17]. The lungs of human beings have higher iron content than the liver, with 0.4 to 0.9 grams of iron per gram of dry weight [18]. The deficiency of iron subsequently leads to anemic conditions. The individuals who were diagnosed with COPD and lower hemoglobin content simultaneously showed poor motor performance [19]. Since the interrelationship between *SERPINA1* and anemia has neither been studied nor been well-established, the novelty of the current findings is significant.

The present findings on the genetic analysis of the study suggest that the polymorphism of *SERPINA1* at rs28929474 and rs17580 are interrelated with the anemic and COPD conditions. Supportive evidence has been found in a recent study that reported that rs28929474 and rs17580 allelic location of *SERPINA1* have an influencing role in lung function [20].

Studies have revealed that alpha 1 antitrypsin, a product of the *SERPINA1* gene, can be used in the treatment of pulmonary disease [21]. The lack of sufficient production of alpha 1 antitrypsin by the *SERPINA1* gene may increase the risk of cancerous growth in the lungs [22]. Studies have discovered that the point mutations in the *SERPINA1* gene, which cause gain of function, lead to cirrhosis, whereas gain of function mutations causes pulmonary emphysema, a clinical condition comprising COPD [23, 24]. Apart from lung disorders, the mutational *SERPINA1* gene increases the risk of inflammation in liver cells, leading to hepatocellular disorders [25].

The genetic polymorphic analysis of the *SERPINA1* gene at the two specific locations rs28929474 and rs17580 gave a positive correlation between anemia, COPD, and mutated *SERPINA1* in the current research work. Apart from these two considered specific locations, another single nucleotide polymorphism may be present in the *SERPINA1* gene since the normal sequence of some anemic and COPD patients is unamplified.

## 5. Conclusion

Various literature studies are available for the correlation of the *SERPINA1* allelic variations at rs28929474 and rs17580 with respiratory diseases, specifically COPD. The current study has revealed the interrelationship between anemia and rs28929474 variation, which is significant, and the considerable role of rs17580 variation in anemic conditions, along with the altitudinal comparison, which is a novel study so far. From the obtained results, it can be concluded that the genetic basis of anemic conditions and COPD is interlinked since the polymorphic presence has been observed commonly for both the diseases and the *SERPINA1* gene underlies the genetic interlink between these two clinical conditions. Anemia is most commonly present without being hereditary. But, contrastingly, the *SERPINA1* gene polymorphism causing an anemic condition may be genetic and may pass through generations. The COPD condition, along with anemia conditions due to genetic factors, worsens the clinical features of the patients and also increases the mortality rate. Hence, it can be concluded that there is a genetic interlink between anemia and COPD with the *SERPINA1* gene in common, as well as altitude has an influencing role in anemic and COPD conditions.

## Figures and Tables

**Table 1:** Sample categorisation.

Altitude	Anemia (*n*)						COPD (*n*)						Control (*n*)					
	<30 years		30 to 50 years		>50 years		<30 years		30 to 50 years		>50 years		<30 years		30 to 50 years		>50 years	
	M	F	M	F	M	F	M	F	M	F	M	F	M	F	M	F	M	F
Low	06	10	22	21	34	10	0	0	08	0	20	24	17	25	13	12	22	14
High	08	10	16	33	08	29	0	0	07	08	20	18	06	31	17	27	13	09
Total	14	20	38	54	42	39	0	0	15	8	40	42	23	56	30	39	35	23
	207						105						206					

COPD: chronic obstructive pulmonary disease; M: male; F: female.

**Table 2:** Polymerase chain reaction—primer and conditions.

*SERPINA1* location	Forward primer (5′→3′)	Reverse primer (5′→3′)	Annealing temperature
Whole sequence	TGAGGCAGACACAACCGTC	GGTTCAGAGAGGTGAAGCGG	54.7 °C
rs28929474	AGACATGGGTATGGCCTCTAA	GCATAAGGCTGTGCTGACCA	56.1 °C
rs17580	GCCATCTTCTTCCTGCCTGAT	CCAGGAACTTGGTGATGATATCGT	58.7 °C

**Table 3:** Number of samples showed positive and negative amplification.

Sample	Nonmutation specific primer		rs28929474		rs17580	
	+(*n*)	–(*n*)	+(*n*)	–(*n*)	+(*n*)	–(*n*)
Anemia—low altitude	46	57	31	26	0	26
Anemia—high altitude	27	77	52	25	2	23
COPD—low altitude	8	44	31	13	2	11
COPD—high altitude	4	49	36	13	10	3
Control—low altitude	102	1	0	1	0	1
Control—high altitude	101	2	0	2	0	2

COPD: chronic obstructive pulmonary disease; +: positive amplification; –: negative amplification.

## Data Availability

The authors confirm that the data supporting the findings of this study are available upon reasonable request with corresponding author.
